# Identification of* Dietzia *spp. from Cardiac Tissue by 16S rRNA PCR in a Patient with Culture-Negative Device-Associated Endocarditis: A Case Report and Review of the Literature

**DOI:** 10.1155/2016/8935052

**Published:** 2016-12-22

**Authors:** Praveen Sudhindra, Guiqing Wang, Robert B. Nadelman

**Affiliations:** ^1^Division of Infectious Diseases, New York Medical College, Valhalla, NY 10595, USA; ^2^Department of Pathology, New York Medical College, Valhalla, NY 10595, USA

## Abstract

The genus* Dietzia* was recently distinguished from other actinomycetes such as* Rhodococcus*. While these organisms are known to be distributed widely in the environment, over the past decade several novel species have been described and isolated from human clinical specimens. Here we describe the identification of* Dietzia natronolimnaea/D. cercidiphylli* by PCR amplification and sequencing of the 16S rRNA encoding gene from cardiac tissue in a patient with culture-negative device-associated endocarditis.

## 1. Introduction

Blood culture-negative endocarditis is a term used to describe definite or probable endocarditis where three or more aerobic and anaerobic blood cultures collected over 48 hours do not yield growth despite prolonged incubation. 2.5% to 31% of all cases of endocarditis end up being culture negative [1]. The wide variation can be accounted for by the use of varying diagnostic and sampling criteria as well as the distribution of fastidious zoonotic agents that are known to cause endocarditis. The use of implantable cardiac devices such as pacemakers and defibrillators have added another dimension to the problem. The rate of device-associated infection has been estimated to be 1.5–2.4% [2]. We describe a case of device-associated culture-negative endocarditis in which a* Rhodococcus*-like organism, recently classified in a separate genus, was identified by a broad range PCR and DNA sequencing of the 16S rRNA gene performed on cardiac tissue.

## 2. Case Report

A 58-year-old man was admitted with a three-month history of daily fevers and night sweats. The fevers were preceded by oral canker sores, all but one of which had resolved at the time of presentation. He also had weight loss that he could not quantify and a poor appetite. He denied any other associated symptoms. An automated implantable cardioverter defibrillator (AICD) had been placed several years earlier for primary prevention of ventricular tachycardia. Two weeks prior to admission, the patient had seen his primary care physician and underwent imaging and blood tests, including two sets of blood cultures that were negative. He was not prescribed any antibiotics. Two days prior to presentation he was seen by his cardiologist, who performed a transesophageal echocardiogram at another hospital. The study revealed global left ventricular dysfunction and mobile echo densities on the atrial aspect of the AICD lead. Additional blood cultures were drawn following which the patient was transferred for further management. Three more sets of blood cultures were drawn after which intravenous vancomycin and gentamicin were administered.

Past medical history was significant for chronic atrial fibrillation that was managed by AV nodal ablation and a pacemaker that had been upgraded to an AICD three years prior to presentation. In addition, he had psoriasis, gout, and obstructive sleep apnea. Home medications included rivaroxaban, allopurinol, amlodipine, atorvastatin, furosemide, carvedilol, lisinopril, alprazolam, meloxicam, and omeprazole. The patient lived with his wife, owned a cable company, and had never smoked or used illicit drugs. He drank alcohol occasionally. He did not have any pets or animal contact. He had travelled extensively throughout the United States, but not in the past year.

On examination the temperature was 100.9°F, blood pressure 125/56 mmHg, pulse 59 beats per minute, and an oxygen saturation 94% while breathing ambient air. There was a solitary 2-3 mm ulcer in the right buccal mucosa with a clean base and mild surrounding erythema. The lungs were clear to auscultation and heart sounds were normal without an audible murmur or rub. The pacemaker pocket on the left upper chest had a healed incision and was not tender or erythematous. There were healing psoriatic lesions on the lower abdomen, back, thighs, and forearms. Examination of the fingertips revealed pitting, but no splinter hemorrhages.

Laboratory data were significant for a leukocyte count of 5,900 cells/mm^3^, hemoglobin of 13.9 g/dL, platelet count of 161,000/mm^3^, and a creatinine of 0.94 mg/dL. Urine dipstick revealed 2+ protein (100 mg/dL). ELISA for antibody to human immunodeficiency virus was negative. Liver function assays were within normal limits. Chest roentgenogram showed an AICD, with clear lungs.

Five days after admission, the patient underwent pacemaker lead extraction, excision of the left atrial appendage, closure of a patent foramen ovale, and epicardial pacemaker lead placement. All blood cultures were negative. Serologies for* Bartonella *spp*., Legionella* spp.,* Brucella* spp., and* Coxiella burnetii* were negative as were fungal blood cultures, as well as bacterial, fungal, and mycobacterial cultures of the vegetation and atrial tissue. Nasopharyngeal swab for respiratory pathogen multiplex PCR (including* Mycoplasma pneumoniae*,* Chlamydophila pneumoniae, *and* Bordetella pertussis*) was negative.

Intravenous ceftriaxone was added to the patient's regimen as he remained intermittently febrile a week after admission. Serum IgA antibody to* C. pneumoniae* was positive at a titer of 1 : 128 as was IgM at a titer of 1 : 64; IgG was negative.

A broad range PCR and DNA sequencing targeting 16S rRNA gene was performed on the excised atrial tissue by using the MicroSEQ 500 16S rDNA Sequencing Kit (Life Technologies, Grand Island, NY). The yielded DNA sequence revealed a 100% identity for* Dietzia natronolimnaea. *A subsequent Basic Local Alignment Search Tool (BLAST) search revealed that* D. cercidiphylli *has an identical 16S rRNA sequence and cannot be distinguished from* D. natronolimnaea* using the MicroSEQ 500 Sequencing Kit. PCR was not performed on the vegetation itself, since there was insufficient sample remaining after cultures were performed. Pathological examination of the atrial tissue revealed mild chronic endocarditis and reactive changes in the myocardium, thus satisfying the Modified Duke's criteria for diagnosing infective endocarditis.

Intravenous vancomycin, gentamicin, and ceftriaxone were continued for an additional 10 days at which time the patient developed acute kidney injury; this was attributed to cephalosporin- related allergic interstitial nephritis. All three antibiotics were discontinued and intravenous daptomycin and ciprofloxacin were administered. The creatinine improved, following which the patient was discharged with instructions to complete a four-week course of intravenous daptomycin and oral ciprofloxacin. Attempts made to contact the patient subsequently in order to follow up on his response to therapy were unsuccessful.

## 3. Discussion

The genus* Dietzia* was first assigned in 1995 to organisms previously classified as rhodococci [13]. They are aerobic, Gram-positive, nonsporing, catalase positive nonacid fast actinomycetes. Since their identification and classification, a number of new species have been isolated from the environment and from human specimens.* Dietzia natronolimnaea* is one of 13* Dietzia* species currently described. It was first isolated from an East African Soda Lake located in the Kenyan Tanzanian Rift Valley. It is alkaliphilic, growing best at a pH of 9.0 (range 6–10) and at salt concentrations up to 10% [13, 14].

The first report implicating* Dietzia* spp. in human disease was published in 1999 (Table 1). It described the isolation of* Dietzia maris* from the blood of an immunocompromised patient with catheter associated septic shock [3]. Subsequently there have been three other case reports implicating* Dietzia maris* in human infection, one associated with a prosthetic hip joint infection, another from the blood of a patient with respiratory failure, and the third from the pericardial fluid of a patient with aortitis [4, 5].* D. maris* has also been isolated from the skin of asymptomatic subjects [8]. Two other species—*Dietzia papillomatosis* and* Dietzia cinnamea*—were recovered from a blood culture and a dog bite wound, respectively [10, 12]. Finally, a case of late pacemaker pocket infection was reported, in which 16S rRNA PCR performed on specimens collected from the pocket revealed* Dietzia* spp.; further identification was not attempted [9].

Two studies retrospectively identified* Dietzia* spp. from human clinical specimens by sequencing the 16S rRNA genes and performing biochemical analysis (CAMP test) on isolates initially classified as* Rhodococcus equi* [15, 16]. The first study identified 8 out of 15 isolates as belonging to the genus* Dietzia*. The sources of the clinical specimens were not mentioned. Two of these were identified as* Dietzia natronolimnaea*. The second study followed a similar methodology to identify 16 human clinical isolates previously classified as* Rhodococcus equi*. Nine of these isolates were identified as* D. natronolimnaea/D. cercidiphylli*, and the investigators were unable to distinguish between the two species since the 16S rRNA gene sequences were identical. The isolates were obtained from a wide range of sites including wounds, vaginal, peritoneal, and lung biopsies, blood cultures, and a heart valve.

Both studies also found that the isolates were susceptible to a wide range of antibiotics, including vancomycin, amikacin, amoxicillin-clavulanic acid, ampicillin, ceftriaxone, clarithromycin, ciprofloxacin, imipenem, and linezolid. Some of the* D. maris* and* D. natronolimnaea* isolates were found to be resistant to trimethoprim/sulfamethoxazole. Susceptibilities were tested using the Etest Diffusion gradient method (AB Biodisk, Solna, Sweden).

The increasing identification of this genus from human clinical specimens probably reflects a combination of rising awareness about these organisms as well as more sensitive microbiological diagnostic tools, such as 16S rRNA sequencing. This technique relies on amplifying and sequencing conserved bacterial 16S rRNA genes, which are then compared to known bacterial nucleotide sequences made available either by the manufacturer (MicroSeq ID 16S rDNA Full Gene Library V1.0, http://www3.appliedbiosystems.com/cms/groups/web/documents/softwaredownloads/cms_234268.pdf) or by other approved sources. In principle, several thousand bacterial species could be identified based on this technique. It is an attractive tool to identify fastidious organisms that are difficult to culture in the lab as well as to clarify the identity of organisms that are seen on special stains of biopsy specimens, but fail to grow when cultured. The method does have limitations, especially in identifying organisms at the species level, since percentage differences in sequences that constitute separate species are not always agreed upon [17, 18].

The most common reason for blood culture-negative endocarditis is the administration of antibiotics prior to obtaining blood cultures [14, 19]. Our patient had at least 4 sets of blood cultures obtained prior to the administration of antibiotics. The blood cultures were drawn on two different occasions about 10 days apart. Since infective endocarditis is known to be associated with continuous, low grade bacteremia, one would have expected to diagnose endocarditis due to most agents known to cause subacute bacterial endocarditis (e.g.,* Streptococci* or* Enterococcus* spp.). The HACEK group of organisms were previously difficult to isolate by routine blood culture methods, but with modern culture techniques these organisms are usually isolated within three to five days [20, 21].

We do not believe that the positive* C. pneumoniae* serology in our patient indicates infection with this organism. Guidelines for diagnosis of acute respiratory tract infections due to* C. pneumoniae* suggest a cutoff of 1 : 16 for IgM titers and 1 : 512 for IgG. IgM antibodies are expected to appear within 2-3 weeks of primary infection, while IgG antibodies may take 6–8 weeks to become detectable [22]. Our patient had been symptomatic for about 12 weeks prior to testing; therefore one would have expected IgG antibodies to be detectable. In addition, PCR performed on a nasopharyngeal swab specimen and the 16S rRNA PCR assay performed on excised atrial tissue were negative for* C. pneumoniae*. The specificity and reproducibility of these serological tests have been questioned, with a high rate of asymptomatic infection and cross-reactivity with serologies for* Mycoplasma*,* Bartonella*, and* Yersinia* [23, 24]. The applicability of these criteria to the diagnosis of endocarditis is also questionable.* C. pneumoniae *was identified on cardiac tissue by PCR in most cases where the diagnosis of endocarditis due to this organism was made [25]. The lack of follow-up serologic testing was a potential limitation in this case. In the unlikely event that* C. pneumoniae* played an etiologic role, the patient was treated with oral ciprofloxacin, which has activity against this organism.

Contamination during specimen collection, transport, and storage are always concerns when atypical organisms are identified from clinical specimens, even more so when nontraditional and highly sensitive microbiological methods are used. Some species of* Dietzia* may be part of human skin flora and in one case may have caused infection of a pacemaker pocket [6, 9]. We cannot rule out the possibility of PCR contamination in view of negative culture results in our patient. No serological test is currently available for detection of antibodies specific to infection with this organism. However, the DNA sequence of a single PCR amplicon from the excised atrial tissue of this patient shared 100% identity with that of a* D. natronolimnaea/D. cercidiphylli *strain.

Pathologic findings satisfied the Modified Duke's criteria for the diagnosis of infective endocarditis, classifying it as “Definite Infective Endocarditis”; however, not all clinical criteria were met since this was a case of culture-negative, device-associated endocarditis. The antimicrobial regimen was selected after considering the then current guideline recommendations by Baddour et al. [26] as well as available clinical and epidemiological data. We also considered the rarity of* Dietzia* infections, relative paucity of information regarding the course of disease caused by these organisms and long term response to antibiotics, as well as the high likelihood that the patient would require a new implantable cardiac device after completing antibiotic therapy. Therefore, we selected a regimen which was likely to be convenient for outpatient therapy, well tolerated, and treat most* Enterococci*,* Staphylococci*,* Streptococci, *and HACEK organisms.

The etiologic role of* Dietzia natronolimnaea*/*D. cercidiphylli *in this case of culture-negative device-associated endocarditis is supported by the multiple reported instances where* Dietzia *spp. were isolated from blood cultures as well as one instance each where organisms were identified from heart valve, aortic wall, and pericardial fluid (Table 1). Potential portals of entry may have been the psoriatic lesions on our patient's skin (cultures of the patient's skin were not performed) or from the pacemaker pocket site following implantation of the AICD.

This case serves to illustrate the potential value of PCR and DNA sequencing in diagnosing culture-negative endocarditis and the potential role of* Dietzia *spp. in causing this infection. Developing serological tests could help in clarifying the role played by these organisms in human disease.

## Figures and Tables

**Table 1:** A PubMed search performed in June 2016 using the terms “Dietzia”, “Dietzia and infection” revealed the following published case reports identifying *Dietzia* spp. from human clinical specimens. This excludes the 24 isolates which retrospectively reclassified organisms initially identified as *Rhodococcus equi.*

Organism (date of publication)	Source
*Dietzia maris* (1999) [3]	Blood culture drawn from in-dwelling catheter and from catheter tip culture
*Dietzia maris* (2001) [4]	Biopsy from site of prosthetic hip joint infection, by 16S rRNA sequencing
*Dietzia maris* (2006) [5]	Culture of specimen from aortic wall and pericardial liquid in a patient with aortitis
*Dietzia maris* (2007) [6]	Skin of 8 healthy human control subjects, by 16S rRNA sequencing
*Dietzia cinnamea* (2006) [7]	Perianal swab culture of bone marrow transplant recipient
*Dietzia papillomatosis* (2008) [8]	Skin culture from a patient with confluent and reticulated papillomatosis
*Dietzia *spp. (2012) [9]	Pacemaker pocket infection, by 16S rRNA sequencing
*Dietzia cinnamea* (2012) [10]	Culture of wound from dog bite
*Dietzia aurantiaca* (2012) [11]	CSF culture
*Dietzia papillomatosis* (2013) [12]	Blood culture in a 2-year-old with fever, rash, and recent VP shunt placement for syringomyelia

## References

[1] Katsouli A., Massad M. G. (2013). Current issues in the diagnosis and management of blood culture-negative infective and non-infective endocarditis. *Annals of Thoracic Surgery*.

[2] Greenspon A. J., Patel J. D., Lau E. (2011). 16-year trends in the infection burden for pacemakers and implantable cardioverter-defibrillators in the United States : 1993 to 2008. *Journal of the American College of Cardiology*.

[3] Bemer-Melchior P., Haloun A., Riegel P., Drugeon H. B. (1999). Bacteremia due to *Dietzia maris* in an immunocompromised patient. *Clinical Infectious Diseases*.

[4] Pidoux O., Argenson J.-N., Jacomo V., Drancourt M. (2001). Molecular identification of a *Dietzia maris* hip prosthesis infection isolate. *Journal of Clinical Microbiology*.

[5] Reyes G., Navarro J.-L., Gamallo C., De Las Cuevas M.-C. (2006). Type A aortic dissection associated with *Dietzia maris*. *Interactive Cardiovascular and Thoracic Surgery*.

[6] Dekio I., Sakamoto M., Hayashi H., Amagai M., Suematsu M., Benno Y. (2007). Characterization of skin microbiota in patients with atopic dermatitis and in normal subjects using 16S rRNA gene-based comprehensive analysis. *Journal of Medical Microbiology*.

[7] Yassin A. F., Hupfer H., Schaal K. P. (2006). *Dietzia cinnamea* sp. nov., a novel species isolated from a perianal swab of a patient with a bone marrow transplant. *International Journal of Systematic and Evolutionary Microbiology*.

[8] Jones A. L., Koerner R. J., Natarajan S., Perry J. D., Goodfellow M. (2008). Dietzia papillomatosis sp. nov., a novel actinomycete isolated from the skin of an immunocompetent patient with confluent and reticulated papillomatosis. *International Journal of Systematic and Evolutionary Microbiology*.

[9] Perkin S., Wilson A., Walker D., McWilliams E. (2012). Dietzia species pacemaker pocket infection: an unusual organism in human infections. *BMJ Case Reports*.

[10] Hirvonen J. J., Lepistö I., Mero S., Kaukoranta S.-S. (2012). First isolation of *Dietzia cinnamea* from a dog bite wound in an adult patient. *Journal of Clinical Microbiology*.

[11] Kämpfer P., Falsen E., Frischmann A., Busse H.-J. (2012). *Dietzia aurantiaca* sp. nov., isolated from a human clinical specimen. *International Journal of Systematic and Evolutionary Microbiology*.

[12] Rammer P., Calum H., Moser C. (2013). *Dietzia papillomatosis* bacteremia. *Journal of Clinical Microbiology*.

[13] Rainey F. A., Klatte S., Kroppenstedt R. M., Stackebrandt E. (1995). *Dietzia*, a new genus including *Dietzia maris* comb. nov., formerly *Rhodococcus maris*. *International Journal of Systematic Bacteriology*.

[14] Koerner R. J., Goodfellow M., Jones A. L. (2009). The genus *Dietzia*: a new home for some known and emerging opportunist pathogens. *FEMS Immunology and Medical Microbiology*.

[15] Niwa H., Lasker B. A., Hinrikson H. P. (2012). Characterization of human clinical isolates of *Dietzia* species previously misidentified as *Rhodococcus equi*. *European Journal of Clinical Microbiology and Infectious Diseases*.

[16] Pilares L., Agüero J., Vázquez-Boland J. A., Martínez-Martínez L., Navas J. (2010). Identification of atypical Rhodococcus-like clinical isolates as *Dietzia spp.* by 16S rRNA gene sequencing. *Journal of Clinical Microbiology*.

[17] Woo P. C. Y., Lau S. K. P., Teng J. L. L., Tse H., Yuen K.-Y. (2008). Then and now: use of 16S rDNA gene sequencing for bacterial identification and discovery of novel bacteria in clinical microbiology laboratories. *Clinical Microbiology and Infection*.

[18] Janda J. M., Abbott S. L. (2007). 16S rRNA gene sequencing for bacterial identification in the diagnostic laboratory: pluses, perils, and pitfalls. *Journal of Clinical Microbiology*.

[19] Slipczuk L., Codolosa J. N., Davila C. D. (2013). Infective endocarditis epidemiology over five decades: a systematic review. *PLoS ONE*.

[20] Apisarnthanarak A., Johnson R. M., Braverman A. C., Dunne W. M., Little J. R. (2002). *Cardiobacterium hominis* bioprosthetic mitral valve endocarditis presenting as septic arthritis. *Diagnostic Microbiology and Infectious Disease*.

[21] Arnold D. M., Smaill F., Warkentin T. E., Christjanson L., Walker I. (2004). *Cardiobacterium hominis* endocarditis associated with very severe thrombocytopenia and platelet autoantibodies. *American Journal of Hematology*.

[22] Kumar S., Hammerschlag M. R. (2007). Acute respiratory infection due to *Chlamydia pneumoniae*: current status of diagnostic methods. *Clinical Infectious Diseases*.

[23] Maurin M., Eb F., Etienne J., Raoult D. (1997). Serological cross-reactions between *Bartonella* and *Chlamydia* species: implications for diagnosis. *Journal of Clinical Microbiology*.

[24] Hyman C. L., Roblin P. M., Gaydos C. A., Quinn T. C., Schachter J., Hammerschlag M. R. (1995). Prevalence of asymptomatic nasopharyngeal carriage of *Chlamydia pneumoniae* in subjectively healthy adults: assessment by polymerase chain reaction-enzyme immunoassay and culture. *Clinical Infectious Diseases*.

[25] Gdoura R., Pereyre S., Frikha I. (2002). Culture-negative endocarditis due to *Chlamydia pneumoniae*. *Journal of Clinical Microbiology*.

[26] Baddour L. M., Wilson W. R., Bayer A. S. (2005). Infective endocarditis: diagnosis, antimicrobial therapy and management of complications. *Circulation*.

